# Amplification of the Chromosomal *bla*_CTX-M-14_ Gene in Escherichia coli Expanding the Spectrum of Resistance under Antimicrobial Pressure

**DOI:** 10.1128/spectrum.00319-22

**Published:** 2022-04-25

**Authors:** Eun-Jeong Yoon, You Jeong Choi, Dokyun Kim, Dongju Won, Jong Rak Choi, Seok Hoon Jeong

**Affiliations:** a Department of Laboratory Medicine, Yonsei University College of Medicine, Seoul, South Korea; b Research Institute of Bacterial Resistance, Yonsei University College of Medicine, Seoul, South Korea; c Division of Antimicrobial Resistance Research, Korea National Institute of Health, Korea Disease Control and Prevention Agency, Cheongju, South Korea; Brown University

**Keywords:** CTX-M ESBL, *Escherichia coli*, gene amplification, transcription level, resistance spectrum

## Abstract

Various forms of adaptive evolution occur in clinical isolates in response to the presence of antimicrobial drugs. Among a total of 171 CTX-M-9 group/family extended-spectrum beta-lactamase (ESBL)-producing Escherichia coli blood isolates recovered between 2016 and 2017 in six general hospitals, 50.3% of the isolates possessed the *bla*_CTX-M-14-like_ gene in their chromosome rather than in a plasmid. Focusing on this unprecedented way of the *bla*_CTX-M_ ESBL gene possession, molecular epidemiology of the isolates was assessed and the chromosomal location of the acquired cephalosporinase gene was dissected in an evolutionary point of view. Taking advantage of a complete collection of E. coli blood isolates from a limited period, clonal relatedness of the E. coli isolates carrying the *bla*_CTX-M-14-like_ gene was clarified and the dominant clone, ST131 H30R, was identified. To control the level of resistance and the resistance spectrum to oxyimino-cephalosporin drugs, transcription level of the *bla*_CTX-M-14-like_ gene was tuned finely through positioning the gene near the chromosomal initiation *dnaA* gene and amplifying numbers of the gene in a chromosome using either the copy-and-paste or the tandem amplification methods. Inconspicuous fitness cost by chromosomal location of the gene and free adjustment of the oxyimino-cephalosporin resistance would urge the dominancy of E. coli clinical isolates harboring the *bla*_CTX-M_ ESBL gene in their chromosome.

**IMPORTANCE** Increasing prevalence of E. coli producing CTX-M ESBL is a major concern in clinical settings because it significantly limits treatment options. Thus, it is important to keep watching current molecular mechanisms of resistance and the scheme for dissemination. Recently, chromosomal locations of the *bla*_CTX-M_ genes are often documented in clinical settings and the bacterial strategies were needed to be dissected in an evolutionary point of view. Both main mechanisms of fine tuning the chromosomal gene expression, bacterial gene amplification either by copy-and-paste or by tandem amplification and positioning the gene near the chromosomal initiation *dnaA* gene, were demonstrated in the study, and the fitness cost by the chromosomal location was evaluated.

## INTRODUCTION

Bloodstream infections caused by extended-spectrum β-lactamase (ESBL)-producing Enterobacterales is a life-threatening infectious disease with limited treatment options (1). The global dominant ESBL family is CTX-M, first identified in the late 1990s (2), and subtype 15 in CTX-M-1 group/family and subtypes 14 and 27 in CTX-M-9 group/family are dominant among Enterobacterales worldwide (3). The global spread of these bacteria is known to be due to the contribution of F-type plasmids carrying the genes (4, 5) and Escherichia coli ST131 H30Rx and H30R clones carrying plasmids harboring the *bla*_CTX-M-15_ gene (6) and the *bla*_CTX-M-14/-27_ gene, respectively (7).

While the CTX-M enzymes are known to be ESBLs in all, resistance phenotype, including the spectrum of resistance to oxyimino-cephalosporins, is differed by the enzyme variant (8). The CTX-M-9 group/family ESBLs primarily hydrolyze cefotaxime, and some variants with alterations at the site responsible for the flexibility of the drug-binding pocket have an ability to inactivate a broader range of oxyimino-cephalosporins, including ceftazidime, while the CTX-M-1 group/family enzymes mostly hydrolyze all range of cephalosporins (9).

Basically, the *bla*_CTX-M_ genes are found in plasmids and chromosomal location of the gene in E. coli clinical isolates has been seldom reported as ST38 isolates in Europe (10) and as ST1081 and ST131 isolates in Japan (11) to carry the *bla*_CTX-M-14_ gene in the chromosome.

In this study, E. coli blood isolates carrying the CTX-M-9 group/family gene were assessed and the chromosomal location of the gene for the fine-tuning of gene expression resulting in an adjustment of the level of cephalosporin resistance allowing the bacteria to survive when they encounter deadly antimicrobial drugs.

## RESULTS

### Molecular epidemiology of the E. coli blood isolates producing CTX-M-9 group/family ESBL.

Through a prospective observational study of 1,492 E. coli bloodstream infection cases occurring in a year through the Global Antimicrobial Resistance Surveillance System in South Korea (12), 31.1% of the blood isolates carried at least one *bla*_CTX-M_ gene for ESBL: 51.4% of the CTX-M ESBL producers carried the CTX-M-1 group/family gene and 45.2% of those had the CTX-M-9 group/family *bla*_CTX-M_ gene and 3.4% of those harbored both genes. As ST131 H30Rx was a global major clone carrying the *bla*_CTX-M-15_ gene, dominance of the E. coli clone having the CTX-M-1 group/family gene was not surprising (12), however alike dominancy of the CTX-M-9 group/family *bla*_CTX-M_ gene-carrying E. coli ST131 H30R among the blood isolates, was a noteworthy epidemiology.

**FIG 1:**
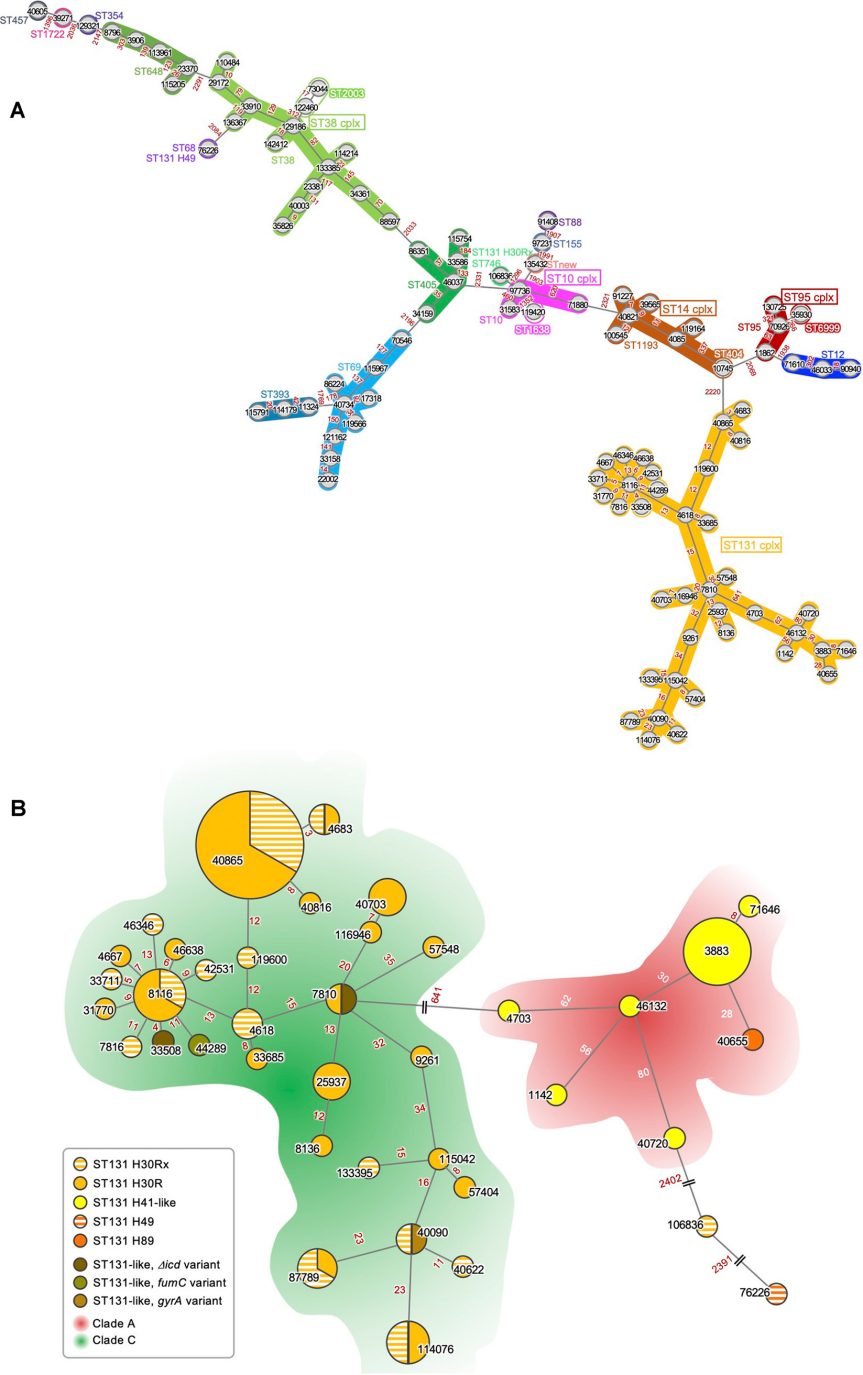
cgMLST-based minimum spanning tree of the E. coli isolates used in the study (A) and those belonging to CC131 (B). The cgSTs are labeled in each circle, and the ST is indicated by the color code. The STs belonging to specific clonal complexes are indicated with unfilled boxes. The numbers near each branch indicate the numbers of different alleles between cgSTs. (B) The number of isolates is associated by the size of each circle on a log scale, and the cgSTs are indicated in each circle.

For the 171 E. coli isolates carrying the CTX-M-9 group/family ESBL gene tested, a total of 25 sequence types (STs) were identified involving five clonal complexes (CCs), including the most identified CC131 (*n* = 90, 52.6%), and 13 singlet STs. In addition, core gene multilocus sequence typing (cgMLST) was carried out for a total of 2,513 core genes. A total of 100 cgSTs were identified (Fig. 1A) and the cgSTs belonging to a ST were gathered together with branch lengths shorter than 2,000, with two exceptional strains belonging to ST131 H49 and ST131 H30Rx in the minimum spanning tree (MST). The MST of cgMLST belonging to the largest CC131 (Fig. 1B) presented that H41-like isolates were clustered apart from H30. Through *in silico* analyses, 42 distinct serotypes were identified, mostly differed by ST, as the mobilomes were (Fig. S1).

### CTX-M-9 group/family *bla*_CTX-M_ ESBL gene-carrying E. coli blood isolates.

From the 171 E. coli isolates, seven variants of CTX-M-9 group/family ESBL were identified. The dominant variant was CTX-M-14 (*n* = 112), and the second most common variant was CTX-M-27 (*n* = 51). Variants CTX-M-27, CTX-M-98, and CTX-M-174 included a D240G substitution conferring expanded resistance to ceftazidime (Fig. S2).

The CTX-M-9 group/family ESBL genes were located either in the chromosome (50.3%, 86/171) or in the plasmid (*n* = 94) and nine isolates harbored copies of this gene not only in their chromosomes but also in their plasmids. Five isolates contained two *bla*_CTX-M-14-like_ gene-carrying plasmids and 20 isolates carried two (*n* = 12), three (*n* = 6), and five (*n* = 2) copies of the *bla*_CTX-M-14_ gene in their chromosomes. Notably, 13 of the 171 isolates also harbored the CTX-M-1 group/family *bla*_CTX-M-15_ gene, and 12 of those carried the gene in their chromosome.

### CTX-M-9 group/family ESBL gene-carrying plasmids.

**FIG 2:**
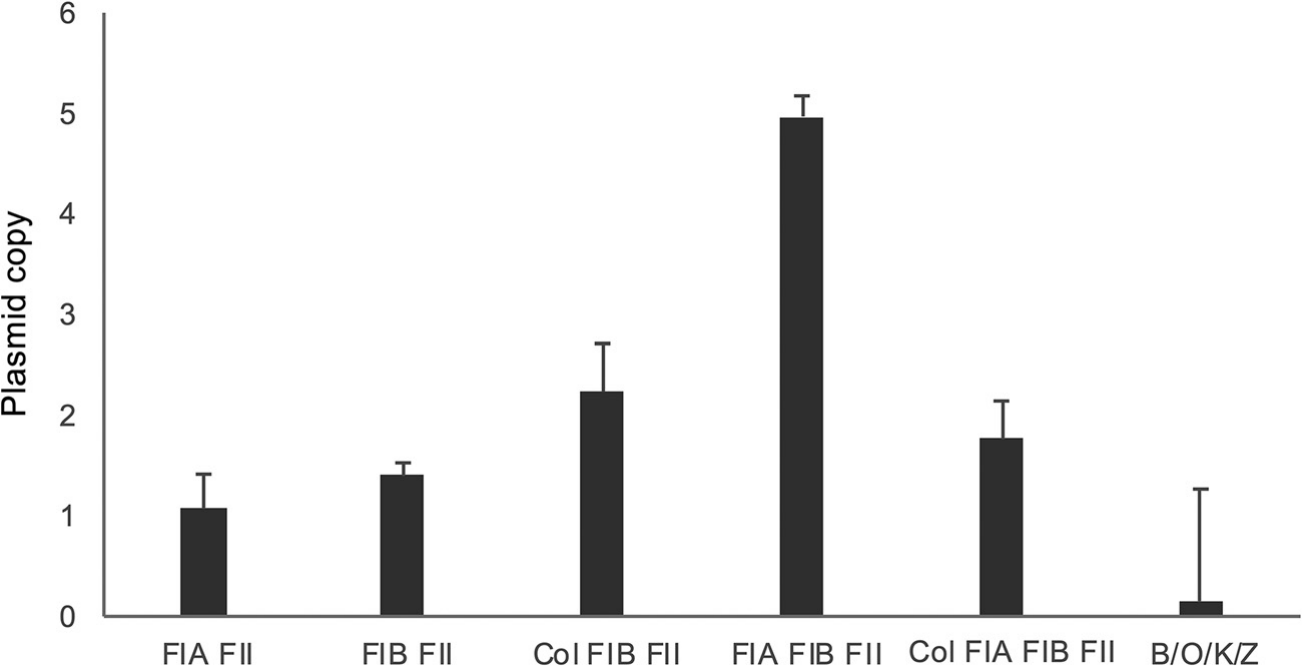
Number of copies of the *bla*_CTX-M-14_-carrying plasmid. For representative *bla*_CTX-M-14_-carrying plasmids, the number or copies per bacterium was determined by using qPCR and is indicated in a bar graph. Gene copies are the average of three independent experiments, and error bars represent the standard deviation.

As five isolates possessed two plasmids carrying the gene, a total of 99 *bla*_CTX-M-14-like_ gene-carrying plasmids carried by 94 isolates were analyzed. The incompatibility group could not be determined for 15 plasmids, and 13 different plasmid incompatibility groups were identified (Table S1). The number of copies of a given plasmid in each bacterium was determined for the representative *bla*_CTX-M-14_ gene-carrying plasmids (Fig. 2): the FIA/FIB/FII plasmid showed the highest number of copies (4.97 ± 1.12), and the B/O/K/Z plasmid showed the lowest (0.15 ± 0.13), while the others exhibited 1.08 to 2.23 copies of the given plasmid per chromosome. The correlation between oxyimino-cephalosporin MICs and the incompatibility type was unnoted.

The stability of *bla*_CTX-M-14_-carrying plasmids was evaluated among six plasmids over a total of 360 generations. The FIA/FIB/FII plasmid carrying the unpaired RelA toxin was lost within 36 generations, while the other plasmids were stable until the end of the experiment.

Transfer efficiency of the *bla*_CTX-M-14_ gene-carrying plasmid was evaluated to the bacterial hosts of five dominant STs among E. coli blood isolates (Table S2): the ST131 often carrying either the *bla*_CTX-M-15_ or the *bla*_CTX-M-14_ gene and other four STs rarely carrying the *bla*_CTX-M_ gene, including ST95, ST69, ST1193, and ST73 (10). The B/O/K/Z plasmid was transferred to various bacterial hosts efficiently, while the Col/FIA/FIB/FII plasmid was untransferable to any bacterial host. The I1-type plasmid was transferred to limited recipients.

### Chromosomal location of the CTX-M-9 group/family genes.

**FIG 3:**
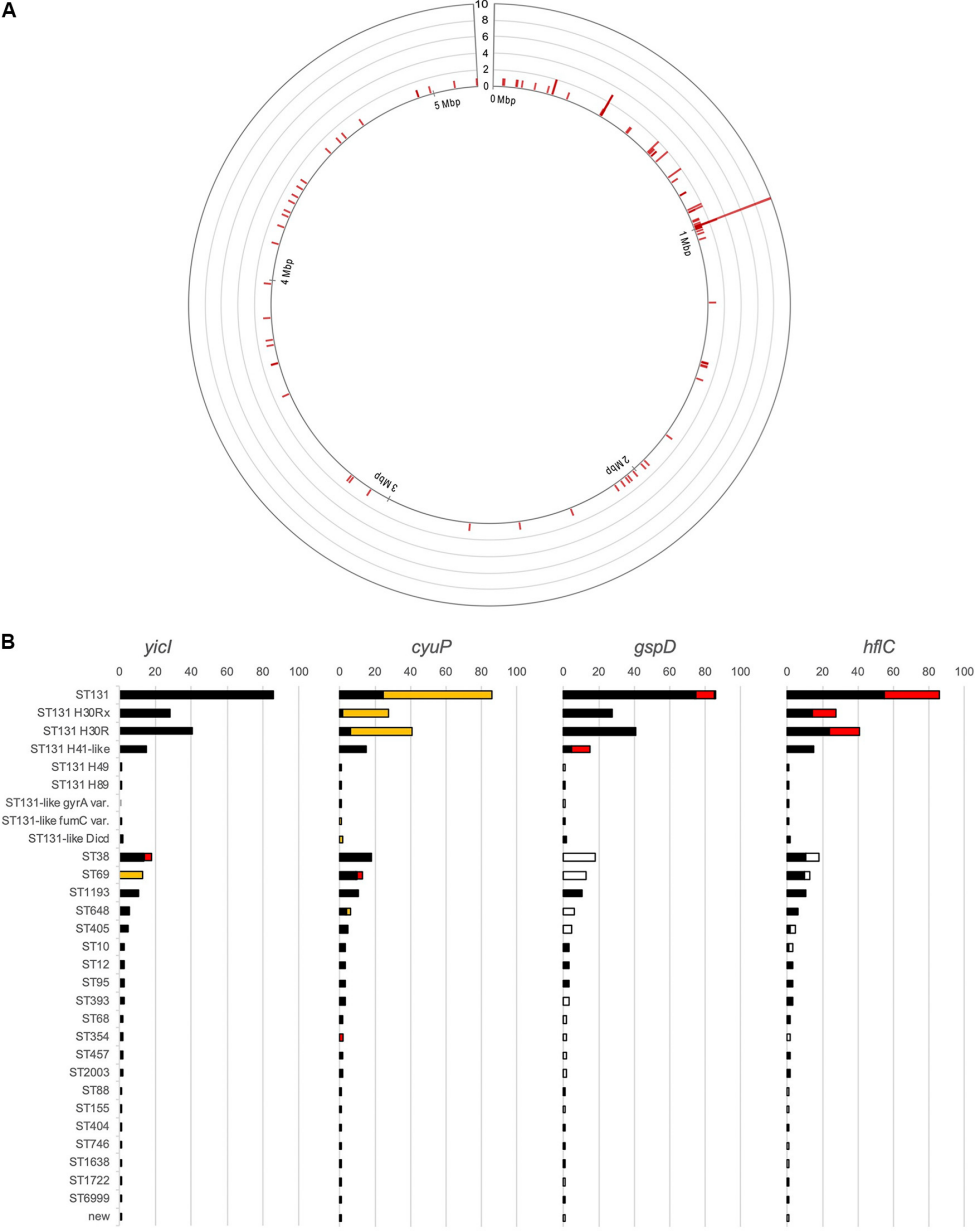
Geographical location of *bla*_CTX-M-14_-like gene integration in the chromosome (A) and Chromosomal integration hot spots of the *bla*_CTX-M-14_ gene (B). (A) The circle represents the chromosome of the E. coli isolate starting with the *dnaA* gene, encoding the chromosomal replication initiator. The red bar indicates the number of chromosomes carrying the gene at the location. Chromosomal rearrangement was not considered in relation to positioning. (B) Present completeness of the four chromosomal genes most commonly interrupted by *bla*_CTX-M-14_-like gene integration. The four hot spots were the *yicI* gene for alpha-d-xyloside xylohydrolase, the *cyuP* gene for cysteine detoxification protein, the *gspD* gene for type II secretion system protein GspD, and the *hflC* gene for regulator of the FtsH protease. The numbers of chromosomes of the E. coli isolates in an ST are indicated by using a bar graph, and each instance of coding sequence (CDS) interruption is indicated by a color code: black, the gene in an intact form; red, the CDS disrupted by the *bla*_CTX-M-14_ gene; yellow, the CDS disrupted by something else, but not the *bla*_CTX-M-14_ gene; white, devoid of the CDS, not only at the appropriate position but also in the rest of the chromosome. The *cypP* gene was most commonly disrupted by the IS*Ec58* element in ST131 isolates. The *yicI* gene was targeted in ST38 isolates, and the gene in ST69 was completely disrupted by the integration of the IS*4* element.

While the first quarter (1q) of the chromosome from the chromosomal initiation *dnaA* gene was most commonly targeted for integration (Fig. 3A), the targets of *bla*_CTX-M_ gene integration were likely random. The consensus sequence was not AT-rich, unlike that of Klebsiella pneumoniae (Fig. S3) (13). Regarding the integration of *bla*_CTX-M-14_, the *hflC* and *gspD* genes were the most commonly targeted genes in ST131 isolates; *cyuP* was the most commonly targeted in ST69; and the *yicI* gene was the most commonly targeted in ST38 isolates (Fig. 3B). *In vitro* integration from the plasmid to the chromosome was not occurred on the bench.

The promoter sequences provided by the 3′-end of the IS*Ecp1* element was identical, and the level of gene expression was correlated with the gene location, especially when the location was within the 1q region (Table S3): the isolates carrying the gene at sites located 34 kb, 625 kb, and 803 kb from the chromosomal initiation site showed *bla*_CTX-M-14_ gene transcription levels 2.38 ± 0.14, 2.03 ± 0.25, and 1.71 ± 0.12, respectively, and cefotaxime MICs ranging from 32 to 16 mg/L reflected the level of *bla*_CTX-M-14_ gene expression similarly to the MICs of ceftzidime and cefepime. In the two isolates in which the gene was located at 2q, the gene transcription levels were 1.78 ± 0.12 and 1.72 ± 0.06, and the MICs of cefotaxime, ceftazidime, and cefepime were 8 mg/L, 0.5 - <0.25 mg/L, and 2 mg/L, respectively. Isolates carrying the *bla*_CTX-M-14_ gene only within the chromosome exhibited MICs of cefotaxime, ceftazidime, and cefepime correlated with the number of gene copies (Fig. S4).

### The *bla*_CTX-M-14_ gene amplification can be induced *in vitro* with oxyimino-cephalosporins.

**FIG 4:**
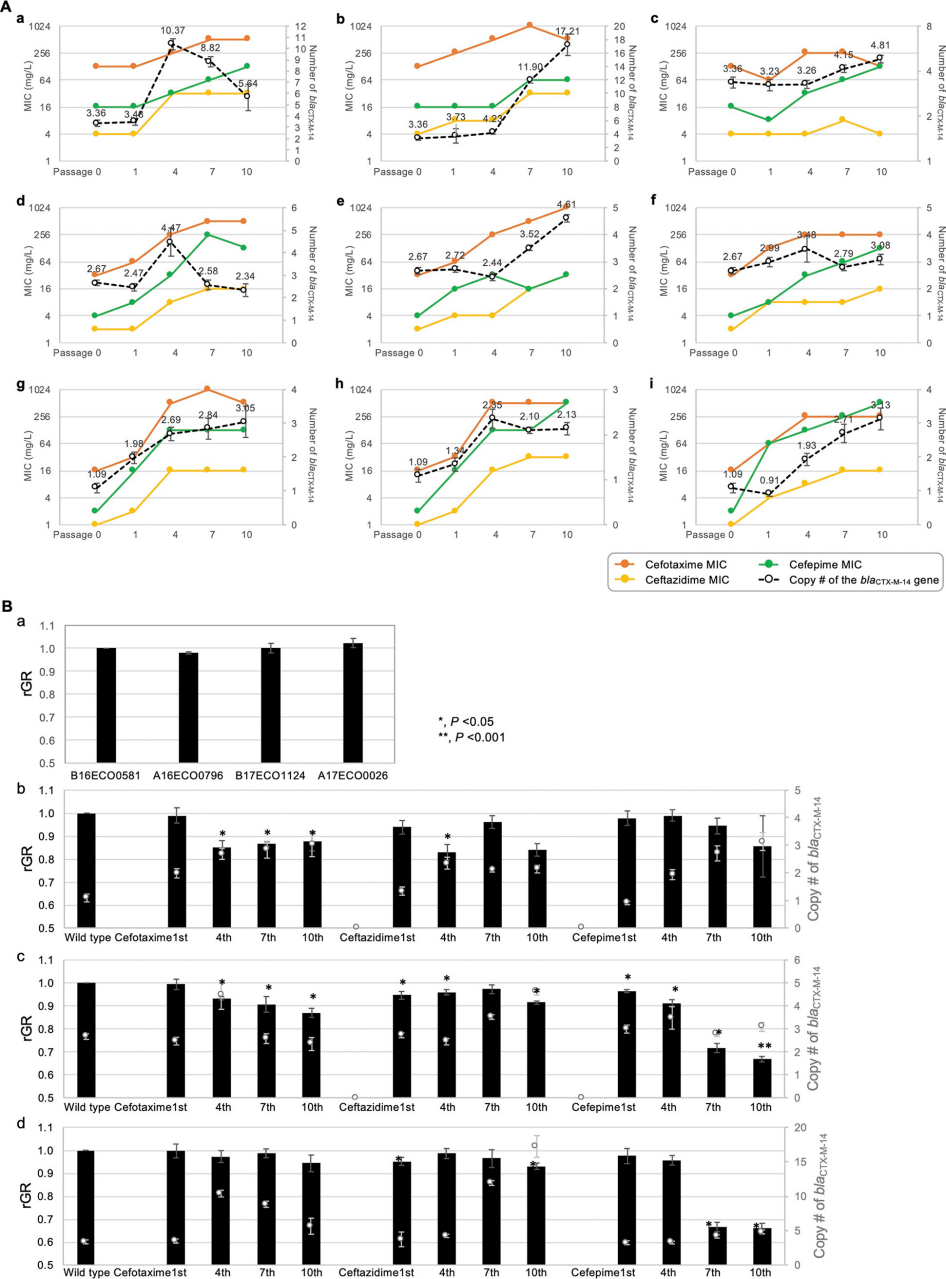
Relative gene copy numbers and MICs of cephalosporin drugs during growth in the presence of cephalosporins (A) and the relative growth rates (B). (A) The E. coli isolates A16ECO0796 (a, b, c), B16ECO1124 (d, e, f), and A17ECO0026 (g, h, i) carrying one, two, and three copies of *bla*_CTX-M-14_ in their chromosomes were grown in MH broth with cefotaxime (a, d, g), ceftazidime (b, e, h), or cefepime (c, f, i) at 0.5× MIC, and the cultures were collected in the initial, first, forth, seventh, and tenth passages. For the collection of four-point passages, the relative number of copies of *bla*_CTX-M-14_ (broken line) was determined by qPCR, and the MICs of cefotaxime (orange line), ceftazidime (yellow line), and cefepime (green line) were determined by broth microdilution methods. The reported gene copies are the average of three independent experiments, and error bars represent the standard deviation. (B) Relative growth rates of the isolates carrying one to three copies of the *bla*_CTX-M-14_ gene in the chromosome versus drug-susceptible B16ECO0581 (A); those of the A16ECO796 subcultures under cephalosporine drug treatments versus the wild-type A16ECO796 isolate (B); those of the A16ECO796 subcultures under cephalosporine drug treatments versus the wild-type A16ECO796 isolate (C); and those of the A16ECO796 subcultures under cephalosporine drug treatments versus the wild-type A16ECO796 isolate (D). The copy numbers of the *bla*_CTX-M-14_ gene determined by qPCR are presented as open gray dots. Statistical significance analyzed by Student's *t* test is indicated with asterisks in each bar graph.

The isolates carrying one to three copies of the *bla*_CTX-M-14_ gene in their chromosomes (A16ECO0796, with a single copy of the gene (796Ω1c); B16ECO1124, with two copies (1124Ω2c); and A17ECO0026, with three copies (26Ω3c)) were subcultured for 10 passages in the presence of 0.5× the MIC of cefotaxime, ceftazidime, or cefepime and the MIC changes were recorded with the estimated *bla*_CTX-M-14_ gene copies by qPCR (Fig. 4A). The 796Ω1c isolate displayed MIC elevation immediately after drug exposure and showed gene amplification induced by cefotaxime and ceftazdime. In contrast, changes induced by cefotaxime and ceftazidime were not observed until the 4th and 7th passages, respectively, in 1124Ω2c and 26Ω3c. Under cefepime treatment, none of the isolates except for 796Ω1c presented robust MIC elevation or gene amplification. The 796Ω1c isolate showed passage-dependent MIC elevation and gene amplification beginning in the 4th passage.

Long-read sequencing-based analysis showed that after the 10th passage under cefotaxime and ceftazidime treatment, 796Ω1c displayed the copying-and-pasting of a 3,097-bp element, including IS*Ecp1*-*bla*_CTX-M-14_-IS*903* at *ca.* 3.5 kb downstream from the primary site; under cefepime treatment, this isolate presented the copying-and-pasting of this element at *ca.* 100-kb-upstream from the primary site. An intermediate circular DNA sequence composed of three IS*Ecp1*-*bla*_CTX-M-14_-IS*903*-*iroN* transposons was observed in the 7th subculture of 796Ω1c under cefotaxime treatment, but it disappeared shortly, in the 10th passage. In the case of 26Ω3c under cefotaxime treatment, a 25,932-bp tandem duplication was observed after the 4th passage, and it became a tandem triplication after the 7th passage.

The analysis of growth rates of the 796Ω1c, 1124Ω2c, and 26Ω3c isolates relative to that of drug-susceptible isolates indicated that the chromosomal location of the *bla*_CTX-M-14_ gene was not costly (Fig. 4B to A); however, the course of gene amplification under antimicrobial pressure seemed costly depending on the mechanism of gene amplification (Fig. 4B). Tandem amplification via a rolling-circle mechanism, which seemed temporal and unstable, was much costly to the host than the copy-and-paste mechanism.

## DISCUSSION

The *bla*_CTX-M-14_ gene, which is the most spread variant in the CTX-M-9 group/family ESBLs, was originally identified in E. coli blood isolates from South Korea in 1995 (14) and it is now globally disseminated in European countries, Asia, and North America (15). In a study, which was conducted in South Korea in 2005 (16), the *bla*_CTX-M-14_ gene-carrying E. coli clinical isolates were composed of diverse STs, three quarters of the genes were carried by plasmids, mostly the IncF-type plasmids, and one third of the *bla*_CTX-M-14_ genes were found in chromosomes of the bacteria. Major changes found in the blood isolates in 2016 include: i) appearance of the dominant E. coli clone, ST131 H30R, carrying the *bla*_CTX-M-14_ gene, ii) diversified incompatibility types of the *bla*_CTX-M-14_ gene-carrying plasmids, and iii) increasingly documented chromosomal location of the *bla*_CTX-M-14_ gene.

Dominancy of the E. coli ST131 carrying the *bla*_CTX-M-14_ gene could be a part of the global trend and the diversified plasmid types assumed to be associated with the rapid mosaicism of plasmids and emerging plasmid of improved conjugation efficiency. However, the increasing chromosomal location of the gene should be noted macroscopically as a part of evolution. Basically, chromosomal location of the gene has an absolute benefit, including segregational stability of the resistance determinant (17). Half of E. coli clinical isolates included in the study adopted a strategy of fine tuning the level of chromosomal gene expression (17), rather than repeated losing and acquiring the resistance-associated plasmids. The CTX-M-14 ESBL confers high-level resistance to cefotaxime but not to ceftazidime (14), and the level of gene expression could result in the expanded spectrum of oxyimino-cephalosporine resistance.

Bacterial gene amplification is one of the key strategies employed by bacteria encountering antimicrobial pressure (18). Limited biological costs relative to plasmid localization (19) is an additional profit for the bacteria carrying the *bla*_CTX-M-14_ gene in their chromosomes. The way of fine tuning the level of chromosomal gene expression includes gene amplification (17) and the location of a gene in a chromosome (20). Both the ways allowed adjustment of the level of resistance, including an expansion of the resistance spectrum. ST131 H30R, which is a notorious clone in clinical settings, most frequently carried the *bla*_CTX-M-14_ gene in the 1q chromosomal region, allowing maximum resistance to antimicrobial drugs. In the 1q region of the chromosome, translation, ribosomal structure and biogenesis-associated protein, transcription-associated protein, posttranslational modification, protein turnover, chaperon-associated protein, and carbohydrate transport and metabolism-associated protein coding genes were more abundant than the other part of the chromosome (Fig. S5), which are essential for the protein biosynthesis and metabolism (20).

In this study, we observed an interesting adaptive evolution occurring in clinical isolates in response to the presence of antimicrobial drugs in addition to the dynamic mechanisms of the emergence and dissemination of antimicrobial resistance (21). Most of the experiments were carried out using clinical isolates with clear resistance geno- and phenotypes. The results imply the impact of the chromosomal location of the resistance gene and highlight the fine-tuning of the gene in isolates encountering life-threatening challenges.

## MATERIALS AND METHODS

### Isolates used in the study.

Among a total of 223 E. coli blood isolates carrying the CTX-M-9 group/family gene that were isolated between May 2016 and April 2017, 52 isolates were screened out through the process of recovering the isolates and the PCR confirmation of the gene, and a total of 171 E. coli blood isolates were finally used in the study. Antimicrobial susceptibility testing to determine the MICs of cefotaxime, ceftazidime, and cefepime was carried out by using the broth microdilution method.

### Whole-genome sequencing.

Total DNA was extracted from each of the 171 E. coli isolates using the GenElute bacterial genomic DNA kit (Sigma–Aldrich, St. Louis, MO). Following the quality control of the extracted DNA, libraries were prepared using the SMRTbell Express Template Prep kit 2.0 (Pacific Biosciences of California, Inc., Menlo Park, CA) following the manufacturer’s instructions. The entire genomes of the isolates were sequenced using PacBio Sequel Systems technology. Reads were assembled using the Microbial Assembly application of SMRT Link v9.0. The annotation of the complete sequences was carried out using prokka 1.13.7 (https://github.com/tseemann/prokka) (22). For important or intriguing genes, Sanger sequencing was carried out for the manually generated PCR amplicons. Genomic DNA was extracted using Cica Geneus DNA extraction reagent (Kanto Chemical Co., Inc., Tokyo, Japan) following the manufacturer’s instructions and was employed for PCR using AccuPower PCR premix (Bioneer, Daejeon, South Korea). Sanger sequencing was then performed to determine the nucleic acid sequences. The primers used for amplification and sequencing are listed in Table S.

### *In silico* molecular epidemiology study using the whole genome.

For multilocus sequence typing (MLST), the allele numbers of seven E. coli housekeeping genes, *adk*, *fumC*, *gyrB*, *icd*, *mdh*, *purA*, and *recA* were determined according to the scheme of Wirth et al. (23) by using MLST 2.0 (24), and the corresponding STs of the isolates were obtained. The cgMLST, implemented in Enterobase (25), was further carried out using a total of 2513 loci. The relatedness of each isolate was inferred by constructing MST using PHYLOViZ (26). The identification of resistant determinants was performed by using ResFinder (https://cge.cbs.dtu.dk//services/ResFinder/). The incompatibility types of the *bla*_CTX-M_ gene-harboring plasmids were determined and plasmid MLST (pMLST) was performed by using plasmid finder (https://cge.cbs.dtu.dk//services/PlasmidFinder/) (27) and pMLST (https://cge.cbs.dtu.dk//services/pMLST/) (28), respectively. Prophages were searched in each genome using the PHAge Search Tool Enhanced Release (PHASTER) database (29). Putative CRISPR estimation was conducted using the associated sequences of *cas* genes in CRISPRFinder (30). Type II toxin/antitoxin systems were searched against the TADB 2.0 database (31), and the subtyping of CTX-M was conducted using an in-house-built database. ST131 subtypes were determined following a set method using the sequence of each genome: H numbering by FimH typing based on an in-house-built database, R typing by ciprofloxacin susceptibility results, and x typing searching for two single nucleotide substitutions based on an in-house-built database.

### Plasmid transfer by bacterial conjugation.

For bacterial conjugation, recipients were generated for the study. Spontaneous mutants were generated from drug-susceptible E. coli clinical isolates devoid of any obvious plasmids by electrophoresis to introduce nucleic acid mutations conferring resistance to both nalidixic acid and sodium azide. Equal amounts of exponential cultures of the donor and recipient isolates were mixed, incubated in Mueller-Hinton broth devoid of any drugs for 12 h, and spread on brain heart infusion agar (Difco Laboratories) containing nalidixic acid (30 mg/L), sodium azide (100 mg/L), and cefotaxime (10 mg/L). Each colony was confirmed by PCR, and the plasmid transfer frequency was calculated as the number of transconjugants per donor. The experiments were performed in duplicate and repeated at least three times.

### Oxyimino-cephalosporin induction of the chromosomal *bla*_CTX-M-14_ gene.

Three E. coli isolates carrying one to three copies of the *bla*_CTX-M-14_ gene in their chromosomes were used in the study. The isolates were trained in the presence of 0.5× the MICs of cefotaxime, ceftazidime, and cefepime, and the trained isolates were stored at −70°C every ca. 36 generations. The stored isolates were recovered at the end of the training, and further experiments were carried out on the different together. The MICs were determined by using broth microdilution methods for cefotaxime, ceftazidime, and cefepime, and copy numbers of the *bla*_CTX-M-14_ gene were determined by quantitative PCR using LightCycler Faststart DNA Master SYBR green I (Roche). For selected isolates, the entire genome was sequenced by using the PacBio sequel system.

### Relative growth rates.

The growth rates of each isolate were determined in microplates coupled to a Multiskan spectrophotometer (Thermo Fisher Scientific, MA) (32). Each isolate was grown overnight at 37°C, and the bacterial cultures were diluted to an optical density at 600 nm (OD_600_) of 0.15 and grown at 37°C with shaking for approximately 2 h. When the cultures reached an OD600 of ca. 0.9, they were diluted 10^−5^-fold, and the diluents were distributed in 96-well microplates at 200 μL per well. During incubation at 37°C with shaking, the absorbance was measured at 590 nm every 3 min. Each culture was replicated three times in the same microplate, and three independent experiments were carried out on three independent days. The growth rates of each isolate were determined at the beginning of the exponential phase, and relative growth rates were calculated as the ratio of the growth rate of the isolate versus that of the wild-type isolate.

### Genome sequence availability.

Genome data for this study are available from National Centers for Bio Informatics in the BioProject under accessions PRJNA782071.
