# Supplementary material for: Amplification of the Chromosomal blaCTX-M-14 Gene in Escherichia coli Expanding the Spectrum of Resistance under Antimicrobial Pressure
Source: Microbiol Spectr. 2022 Apr 25;10(3):e00319-22. doi: 10.1128/spectrum.00319-22 (PMC9241692; doi:10.1128/spectrum.00319-22)
Supplement: SUPPLEMENTAL FILE 1 — Supplemental material. Download spectrum.00319-22-s0001.pdf, PDF file, 1.7 MB [file spectrum.00319-22-s0001.pdf]

Supplemental Materials for

Amplification of the chromosomal *bla*$_{CTX-M-14}$ gene in *Escherichia coli* expanding the spectrum of resistance under antimicrobial pressure

Eun-Jeong Yoon, You Jeong Choi, Dokyun Kim, Dongju Won, Jong Rak Choi, Seok Hoon Jeong*

***Correspondence to : Seok Hoon JEONG**

E-mail: kscpjsh@yuhs.ac

The file includes:

Table S1 to S3

FIG S1 to S5

Table S1. Incompatibility types of the CTX-M-9 group/family gene-carrying plasmids possessed by 94 *E. coli* isolates

MLST	B/O/K/Z	B/O/K/Z/FIA/FIB	Col/FIA/FIB	Col/FIA/FIB/FII	Col/FIB/FII	FIA/FIB	FIA/FIB/FII	FIA/FII	FIB/FII	FII	FIA	HI	I1	ND	Total
ST10	0	0	0	0	0	0	1	0	0	0	0	0	1	0	2
ST12	0	0	0	0	0	0	0	0	0	0	0	0	1	0	1
ST38	1	0	0	0	0	0	0	0	4	1	0	0	0	0	6
ST68	0	0	0	0	0	0	1	0	0	0	0	0	0	0	2
ST69	0	0	0	0	4	0	0	0	0	1	0	0	0	4	10
ST95	1	0	0	0	0	0	0	0	0	0	0	1	1	1	4
ST131	8	0	0	13	2	0	5	1	1	3	1	0	3	7	44
H30R	*4*	*0*	*0*	*9*	*0*	*0*	*2*	*1*	*0*	*2*	*1*	*0*	*2*	*4*	*25*
H30Rx	*3*	*0*	*0*	*4*	*2*	*0*	*3*	*0*	*0*	*1*	*0*	*0*	*0*	*1*	*14*
H41	*1*	*0*	*0*	*0*	*0*	*0*	*0*	*0*	*1*	*0*	*0*	*0*	*0*	*2*	*4*
H49	*0*	*0*	*0*	*0*	*0*	*0*	*0*	*0*	*0*	*0*	*0*	*0*	*1*	*0*	*1*
ST131-like	0	0	0	3	1	0	0	0	0	0	0	0	0	0	4
ST155	0	0	0	0	0	0	0	0	0	0	0	0	1	0	1
ST393	0	0	0	0	1	1	1	0	0	0	0	0	0	0	3
ST404	0	0	0	0	0	0	0	0	0	0	0	0	0	1	1
ST405	1	0	0	0	1	0	0	0	0	0	0	0	0	0	2
ST457	0	0	0	1	0	0	0	0	0	0	0	0	0	1	1
ST746	1	0	0	0	0	0	0	0	0	0	0	0	0	0	1
ST1193	2	1	6	0	0	1	0	0	0	0	0	0	0	1	11
ST1722	0	0	0	0	1	0	0	0	0	0	0	0	0	0	1
ST2003	0	0	0	0	0	0	2	0	0	0	0	0	0	0	2
ST6999	1	0	0	0	0	0	0	0	0	0	0	0	0	0	1
novel ST	0	0	0	0	0	0	0	0	0	0	0	0	1	0	1
Total	16	1	6	17	10	2	11	1	5	5	1	1	8	15	99

Table S2. Plasmid transfer efficiency

	Doner	A17EC0191		A16EC0070		A16EC0321	
	Plasmid	B/O/K/Z		Col FIA FIB FII<br>IncF RST [F1:A2:B20]		IncI1<br>pMLST 166	
Recipient type	Isolate	Per donor	Per recipient	Per donor	Per recipient	Per donor	Per recipient
ST73	D16EC0187	1.3 x 10^{-3} - 3.0 x 10^{-2}	4.8 x 10^{-5} - 1.0 x 10^{-4}	<1.0 x 10^{-9}	<1.0 x 10^{-9}	<1.0 x 10^{-9}	<1.0 x 10^{-9}
	E17EC0289	6.8 x 10^{-5} - 1.5 x 10^{-1}	1.9 x 10^{-7} - 8.5 x 10^{-2}	<1.0 x 10^{-9}	<1.0 x 10^{-9}	1.4 x 10^{-3} - 9.2 x 10^{-2}	5.5 x 10^{-3} - 7.0 x 10^{-3}
	E17EC0299	6.5 x 10^{-5} - 8.8 x 10^{-1}	2.0 x 10^{-7} - 6.6 x 10^{-1}	<1.0 x 10^{-9}	<1.0 x 10^{-9}	2.0 x 10^{-5} - 2.7 x 10^{-3}	1.0 x 10^{-4} - 7.5 x 10^{-3}
ST131 non-H30	B16EC0673	4.3 x 10^{-3} - 2.5 x 10^{-2}	1.4 x 10^{-5} - 1.1 x 10^{-3}	<1.0 x 10^{-9}	<1.0 x 10^{-9}	2.6 x 10^{-5} - 6.1 x 10^{-4}	4.8 x 10^{-5} - 2.1 x 10^{-4}
	E17EC0227	<1.0 x 10^{-9}	<1.0 x 10^{-9}	<1.0 x 10^{-9}	<1.0 x 10^{-9}	<1.0 x 10^{-9}	<1.0 x 10^{-9}
	D17EC0125	9.0 x 10^{-2} - 1.8 x 10^{-1}	2.7 x 10^{-2} - 5.8 x 10^{-1}	<1.0 x 10^{-9}	<1.0 x 10^{-9}	<1.0 x 10^{-9}	<1.0 x 10^{-9}
ST131 H30	B16EC0521	7.4 x 10^{-3} - 8.5 x 10^{-2}	9.4 x 10^{-4} - 5.7 x 10^{-3}	<1.0 x 10^{-9}	<1.0 x 10^{-9}	<1.0 x 10^{-9}	<1.0 x 10^{-9}
	C16EC0546	1.5 x 10^{-1} – 2.3 x 10^{-1}	1.6 x 10^{-1} - 1.9 x 10^{-1}	<1.0 x 10^{-9}	<1.0 x 10^{-9}	<1.0 x 10^{-9}	<1.0 x 10^{-9}
	D17EC0143	2.0 x 10^{-2} - 5.1 x 10^{-2}	4.5 x 10^{-4} - 1.7 x 10^{-3}	<1.0 x 10^{-9}	<1.0 x 10^{-9}	<1.0 x 10^{-9}	<1.0 x 10^{-9}
ST131 H30R	A16EC0581	<1.0 x 10^{-9}	<1.0 x 10^{-9}	<1.0 x 10^{-9}	<1.0 x 10^{-9}	2.6 x 10^{-2} - 4.9 x 10^{-1}	1.5 x 10^{-2} - 2.5 x 10^{-1}
	E17EC0271	2.5 x 10^{-2} - 4.5 x 10^{-1}	7.0 x 10^{-3} - 2.8 x 10^{-2}	<1.0 x 10^{-9}	<1.0 x 10^{-9}	<1.0 x 10^{-9}	<1.0 x 10^{-9}
	F16EC0031	3.0 x 10^{-1} - 5.9 x 10^{-1}	1.0 x 10^{-1} - 2.0 x 10^{-1}	<1.0 x 10^{-9}	<1.0 x 10^{-9}	<1.0 x 10^{-9}	<1.0 x 10^{-9}
ST131 H30Rx	B17EC0046	2.5 x 10^{-5} - 9.7 x 10^{-5}	9.1 x 10^{-2} - 2.9 x 10^{-1}	<1.0 x 10^{-9}	<1.0 x 10^{-9}	<1.0 x 10^{-9}	<1.0 x 10^{-9}
	D16EC0374	2.2 x 10^{-1} - 3.3 x 10^{-1}	5.7 x 10^{-3} - 9.1 x 10^{-2}	<1.0 x 10^{-9}	<1.0 x 10^{-9}	2.6 x 10^{-5} - 2.6 x 10^{-4}	2.3 x 10^{-5} - 3.5 x 10^{-5}
	F16EC0590	<1.0 x 10^{-9}	<1.0 x 10^{-9}	<1.0 x 10^{-9}	<1.0 x 10^{-9}	<1.0 x 10^{-9}	<1.0 x 10^{-9}
ST1193	B16EC0375	4.9 x 10^{-2} - 8.6 x 10^{-1}	1.2 x 10^{-3} - 2.8 x 10^{-2}	<1.0 x 10^{-9}	<1.0 x 10^{-9}	<1.0 x 10^{-9}	<1.0 x 10^{-9}
	C16EC0234	8.8 x 10^{-2} - 3.8 x 10^{-1}	3.5 x 10^{-3} - 1.4 x 10^{-2}	<1.0 x 10^{-9}	<1.0 x 10^{-9}	<1.0 x 10^{-9}	<1.0 x 10^{-9}
ST95	B16EC0225	1.2 x 10^{-1} - 3.8 x 10^{-1}	2.2 x 10^{-3} - 5.9 x 10^{-2}	<1.0 x 10^{-9}	<1.0 x 10^{-9}	<1.0 x 10^{-9}	<1.0 x 10^{-9}
	F17EC0018	4.7 x 10^{-2} - 6.0 x 10^{-2}	9.9 x 10^{-4} - 7.4 x 10^{-3}	<1.0 x 10^{-9}	<1.0 x 10^{-9}	<1.0 x 10^{-9}	<1.0 x 10^{-9}
ST69	B16EC0073	6.0 x 10^{-1} - 2.2 x 10^{-1}	6.5 x 10^{-4} - 3.9 x 10^{-3}	<1.0 x 10^{-9}	<1.0 x 10^{-9}	<1.0 x 10^{-9}	<1.0 x 10^{-9}
	E16EC0858	6.1 x 10^{-2} - 3.2 x 10^{-1}	2.2 x 10^{-4} - 3.5 x 10^{-4}	<1.0 x 10^{-9}	<1.0 x 10^{-9}	7.0 x 10^{-4} - 1.2 x 10^{-2}	5.0 x 10^{-5} - 1.1 x 10^{-4}
	F16EC0563	<1.0 x 10^{-9}	<1.0 x 10^{-9}	<1.0 x 10^{-9}	<1.0 x 10^{-9}	<1.0 x 10^{-9}	<1.0 x 10^{-9}

Table S3. ***bla*$_{CTX-M-14}$ gene transcription levels in *E. coli* blood isolate strains carrying the gene in the chromosome at different locations**

Strain[a]	Location of the gene[b] (bp)	Transcription level[c]	MIC (mg/L)[d]		
			Cefotaxime	Ceftazidime	Cefepime
B17EC0231	31,615	2.38 ± 0.14	32	1	8
B16EC0630	624,649	2.03 ± 0.25	16	0.5	4
B17EC0295	802,981	1.71 ± 0.12	16	0.5	4
C16EC0166	1,377,914	1.78 ± 0.12	8	0.5	2
C16EC0626	1,709,542	1.72 ± 0.06	8	<0.25	2

[a]all the strains had a single *bla*$_{CTX-M-14}$ gene in the chromosome, and no additional acquired genes for cephalosporinases were identified in the genome of each strain.

[b]The location is indicated in bp from the dnaA gene for the initiation of the chromosome.

[c]Transcription level of the *bla*$_{CTX-M-14}$ gene was determined by using RT qPCR relative to the level of *dnaA* gene expression.

[d]The MICs were determined by using broth microdilution methods.

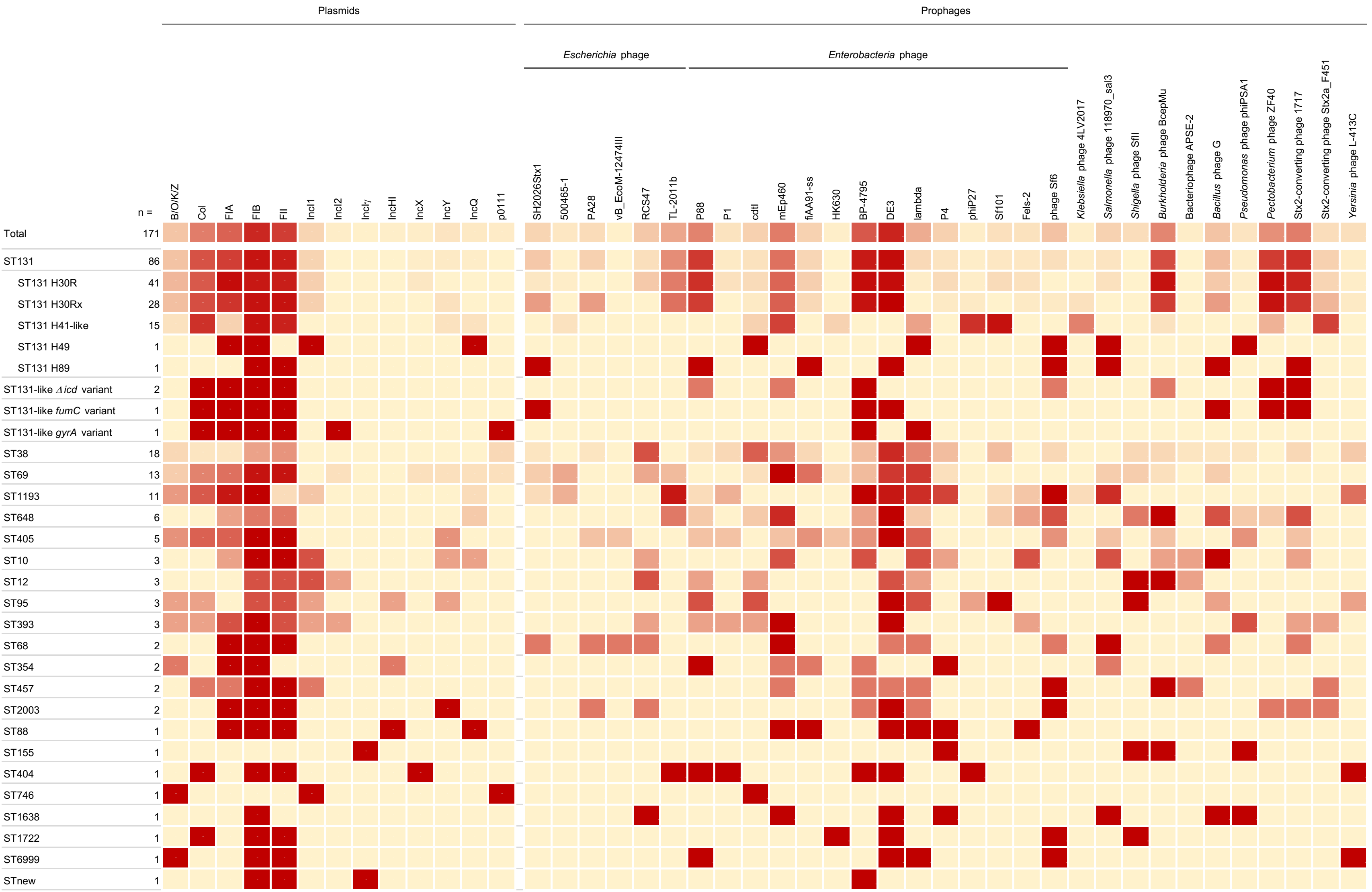

Plasmids
Prophages
Escherichia phage
Enterobacteria phage
n =
B/O/K/Z
Col
FIA
FIB
FII
IncI1
IncI2
Incγ
IncHI
IncX
IncY
IncQ
p0111
SH2026Stx1
500465-1
PA28
vB_EcoM-12474III
RCS47
TL-2011b
P88
P1
cdtI
mEp460
fiAA91-ss
HK630
BP-4795
DE3
lambda
P4
phiP27
Sf101
Fels-2
phage Sf6
Klebsiella phage 4LV2017
Salmonella phage 118970_sal3
Shigella phage SfII
Burkholderia phage BcepMu
Bacteriophage APSE-2
Bacillus phage G
Pseudomonas phage phiPSA1
Pectobacterium phage ZF40
Stx2-converting phage 1717
Stx2-converting phage Stx2a_F451
Yersinia phage L-413C
Total 171
ST131 86
ST131 H30R 41
ST131 H30Rx 28
ST131 H41-like 15
ST131 H49 1
ST131 H89 1
ST131-like Δicd variant 2
ST131-like fumC variant 1
ST131-like gyrA variant 1
ST38 18
ST69 13
ST1193 11
ST648 6
ST405 5
ST10 3
ST12 3
ST95 3
ST393 3
ST68 2
ST354 2
ST457 2
ST2003 2
ST88 1
ST155 1
ST404 1
ST746 1
ST1638 1
ST1722 1
ST6999 1
STnew 1

FIG S1. Heatmaps of the plasmidome and the prophages identified in the isolates of each ST. For the genomes of all tested *E. coli* isolates, plasmid incompatibility typing was carried out in silico, and the presence of prophages was searched in silico by using the PHATER database. The percentage of the genome belonging to the ST was displayed in a heatmap; 100% of isolates indicated by dark red, and 0% of isolates indicated by light yellow.

```
CTX-M-65        MVTKRVQRMMFAAAACIPLLLGSAPLYAQTSAVQQKLAALEKSSGGRLGVALIDTADNTQ        60
CTX-M-24        MVTKRVQRMMFAAAACIPLLLGSAPLYAQTSAVQQKLAALEKSSGGRLGVALIDTADNTQ        60
CTX-M-174       MVTKRVLRMMFAAAACIPLLLGSAPLYAQTSAVQQKLAALEKSSGGRLGVALIDTADNTQ        60
CTX-M-140       MVTKRVQRMMFAAAACIPLLLGSAPLYAQTSAVQQKLAALEKSSGGRLGVALIDTADNTQ        60
CTX-M-98        MVTKRVQRMMFAAAACIPLLLGSAPLYAQTSAVQQKLAALEKSSGGRLGVALIDTADNTQ        60
CTX-M-14        MVTKRVQRMMFAAAACIPLLLGSAPLYAQTSAVQQKLAALEKSSGGRLGVALIDTADNTQ        60
CTX-M-27        MVTKRVQRMMFAAAACIPLLLGSAPLYAQTSAVQQKLAALEKSSGGRLGVALIDTADNTQ        60
                ****** *****************************************************

CTX-M-65        VLYRGDERFPMCSTSKVMAVAAVLKQSETQKQLLNQPVEIKPADLVNYNPIAEKHVNGTM        120
CTX-M-24        VLYRGDERFPMCSTSKVMAAAAVLKQSETQKQLLNQPVEIKPADLVNYNPIAEKHVNGTM        120
CTX-M-174       VLYRGDERFPMCSTSKVMAAAAVLKQSETQKQLLNQPVEIKPADLVNYNPIAEKHVNGTM        120
CTX-M-140       VLYRGDERFPMCSTSKVMAAAAVLKQSETQKQLLNQPVEIKPADLVNYNPITEKHVNGTM        120
CTX-M-98        VLYRGDERFPMCSTSKVMAVAAVLKQSETQKQLLNQPVEIKPADLVNYNPIAEKHVNGTM        120
CTX-M-14        VLYRGDERFPMCSTSKVMAAAAVLKQSETQKQLLNQPVEIKPADLVNYNPIAEKHVNGTM        120
CTX-M-27        VLYRGDERFPMCSTSKVMAAAAVLKQSETQKQLLNQPVEIKPADLVNYNPIAEKHVNGTM        120
                *******************.*******************************:********

CTX-M-65        TLAELSAAALQYSDNTAMNKLIAQLGGPGGVTAFARAIGDETFRLDRTEPTLNTAIPGDP        180
CTX-M-24        TLAELSAAALQYSDNTAMNKLIAQLGGPGGVTAFARAIGDETFRLDRTEPTLNTAIPGDP        180
CTX-M-174       TLAELSAAALQYSDNTAMNKLIAQLGGPGGVTAFARAIGDETFRLDRTEPTLNTAIPGDP        180
CTX-M-140       TLAELSAAALQYSDNTAMNKLIAQLGGPGGVTAFARAIGDETFRLDRTEPTLNTAIPGDP        180
CTX-M-98        TLAELSAAALQYSDNTAMNKLIAQLGGPGGVTAFARAIGDETFRLDRTEPTLNTAIPGDP        180
CTX-M-14        TLAELSAAALQYSDNTAMNKLIAQLGGPGGVTAFARAIGDETFRLDRTEPTLNTAIPGDP        180
CTX-M-27        TLAELSAAALQYSDNTAMNKLIAQLGGPGGVTAFARAIGDETFRLDRTEPTLNTAIPGDP        180
                ************************************************************

CTX-M-65        RDTTTPRAMAQTLRQLTLGHALGETQRAQLVTWLKGNTTGAASIRAGLPTSWTVGDKTGS        240
CTX-M-24        RDTTTPRAMAQTLRQLTLGHALGETQRAQLVTWLKGNTTGAASIRAGLPTSWTVGDKTGS        240
CTX-M-174       RDTTTPRAMAQTLRQLTLGHALGETQRAQLVTWLKGNTTGAASIRAGLPTSWTVGDKTGS        240
CTX-M-140       RDTTTPRAMAQTLRQLTLGHALGETQRAQLVTWLKGNTTGAASIRAGLPTSWTVGDKTGS        240
CTX-M-98        RDTTTPRAMAQTLRQLTLGHALGETQRAQLVTWLKGNTTGAASIRAGLPTSWTVGDKTGS        240
CTX-M-14        RDTTTPRAMAQTLRQLTLGHALGETQRAQLVTWLKGNTTGAASIRAGLPTSWTVGDKTGS        240
CTX-M-27        RDTTTPRAMAQTLRQLTLGHALGETQRAQLVTWLKGNTTGAASIRAGLPTSWTVGDKTGS        240
                ************************************************************

CTX-M-65        GDYGTTNDIAVIWPQGRAPLVLVTYFTQPQQNAERRRDVLASAARIIAEGL        291
CTX-M-24        GDYGTTNDIAVIWPQGRAPLVLVTYFTQPQQNAERRRDVLASAARIIAEGL        291
CTX-M-174       GGYGTTNDIAVIWPQGRAPLVLVTYFTQPQQNAESRRDVLASAARIIAEGL        291
CTX-M-140       GDYGTTNDIAVIWPQGRAPLVLVTYFTQPQQNAESRRDVLASAARIIAEGL        291
CTX-M-98        GGYGTTNDIAVIWPQGRAPLVLVTYFTQPQQNAESRRDVLASAARIIAEGL        291
CTX-M-14        GDYGTTNDIAVIWPQGRAPLVLVTYFTQPQQNAESRRDVLASAARIIAEGL        291
CTX-M-27        GGYGTTNDIAVIWPQGRAPLVLVTYFTQPQQNAESRRDVLASAARIIAEGL        291
                *.******************************** ****************
```

FIG S2. Multiple sequence alignment of the amino acid sequences of the CTX-M-9 group/family ESBLs identified in this study. Aligned sequences are indicated with amino acid counts. The subtypes in red indicate the subtypes showing the D240G substitution conferring resistance to ceftazidime, and the different amino acids compared with CTX-M-14 are indicated in yellow. The signs under the alignment indicate the identity and similarity of each amino acid: asterisks, identical amino acids for all subtypes; dots, diverse amino acids with similar characteristics; colon, diverse amino acids with very similar characteristics; empty, diverse amino acids devoid of similarity.

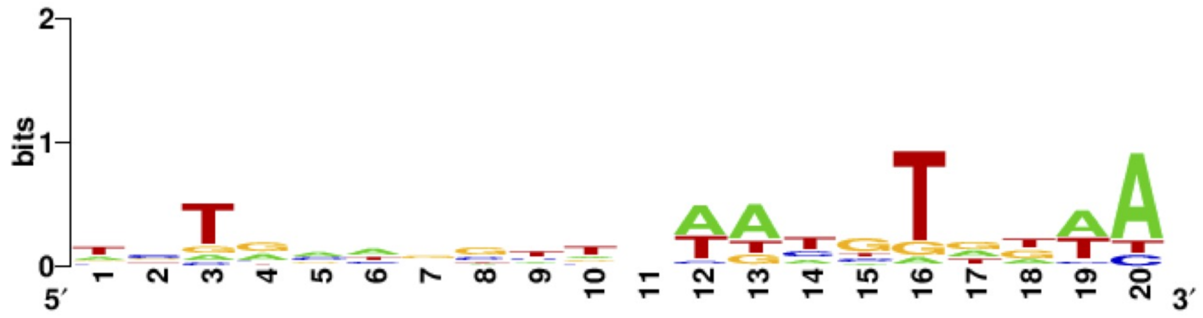

FIG S3. Consensus nucleic acid sequence upstream and downstream of the integration site. Ten aligned nucleotides upstream and downstream of the integration sites were collected and displayed by using WebLogo©.

Cefotaxime MIC (mg/L)	Copy/chromosome 1	2	3	5	Total
4	3				3
8	6				6
16	24				24
32	10	6			16
64	3	3	3		9
128			3	2	5
Total	46	9	6	2	63

Ceftazidime MIC (mg/L)	Copy/chromosome 1	2	3	5	Total
<0.25	5				5
0.5	14	1			15
1	21	3			24
2	2	5	3		10
4	3		2	2	7
8	1		1		2
Total	46	9	6	2	63

Cefepime MIC (mg/L)	Copy/chromosome 1	2	3	5	Total
0.5	2				2
1	4				4
2	12				12
4	20	2			22
8	8	5	3		16
16		2	3	2	7
Total	46	9	6	2	63

FIG S4. Heatmaps of the proportions of isolates harboring different copy numbers of the *bla*$_{CTX-M-14}$ gene in their chromosomes under each MIC of cefotaxime, ceftazidime, and cefepime. In a total of 63 *E. coli* isolates with one to five copies of the *bla*$_{CTX-M-14}$ gene in their chromosome, the MICs of three cephalosporins were determined, and the proportion of isolates at the MIC value was displayed in a heatmap. Darker red indicates a higher proportion of isolates, and lighter red represents a lower proportion of isolates.

COG Class	No. of families	Function	Total abundance	q1	q2	q3	q4
Information storage and processing							
J	245	Translation, ribosomal structure and biogenesis	0.0468	0.0791	0.0278	0.0324	0.0455
K	231	Transcription	0.0791	0.0896	0.0621	0.0696	0.0942
A	25	RNA processing and modification	0.0004	0.0004	0.0000	0.0000	0.0010
L	238	Replication, recombination and repair	0.0571	0.0531	0.0568	0.0652	0.0545
B	19	Chromatin structure and dynamics	0.0000	0.0000	0.0000	0.0000	0.0000
Cellular process and signaling							
O	203	Posttranslational modification, protein turnover, chaperones	0.0362	0.0424	0.0351	0.0379	0.0291
W	1	Extracellular structures	0.0003	0.0010	0.0000	0.0000	0.0000
U	158	Intracellular trafficking, secretion, and vesicular transport	0.0369	0.0421	0.0373	0.0222	0.0471
T	152	Signal transduction mechanisms	0.0525	0.0447	0.0722	0.0540	0.0396
M	188	Cell wall/membrane/envelope biogenesis	0.0620	0.0521	0.0657	0.0632	0.0677
N	96	Cell motility	0.0342	0.0196	0.0435	0.0175	0.0570
V	46	Defense mechanisms	0.0146	0.0100	0.0092	0.0228	0.0167
D	72	Cell cycle control, cell division, chromosome partitioning	0.0086	0.0076	0.0040	0.0143	0.0086
Y	2	Nuclear structure	0.0000	0.0000	0.0000	0.0000	0.0000
Z	12	Cytoskeleton	0.0000	0.0000	0.0000	0.0000	0.0000
Metabolism							
G	230	Carbohydrate transport and metabolism	0.1153	0.1519	0.1087	0.0814	0.1166
E	270	Amino acid transport and metabolism	0.1001	0.1044	0.1067	0.0873	0.1011
C	258	Energy production and conversion	0.0762	0.0721	0.0882	0.0791	0.0664
F	95	Nucleotide transport and metabolism	0.0254	0.0209	0.0278	0.0251	0.0282
Q	88	Secondary metabolites biosynthesis, transport and catabolism	0.0202	0.0128	0.0307	0.0267	0.0111
H	179	Coenzyme transport and metabolism	0.0443	0.0343	0.0313	0.0577	0.0549
I	94	Lipid transport and metabolism	0.0281	0.0205	0.0267	0.0332	0.0327
P	212	Inorganic ion transport and metabolism	0.0649	0.0576	0.0536	0.0739	0.0753
Poorly characterized							
R	702	General function prediction only	0.1242	0.1166	0.1436	0.1451	0.0918
S	1347	Function unknown	0.0977	0.0904	0.0992	0.1050	0.0967

Above the total abundance
Around the total abundance
Below the total abundance
ND in the entire chromosome

Figure S5. Distribution of the coding sequences in the chromosome classified through the functional annotation using the Clusters of Orthologous Groups (COGs) protein database. The functional annotation was conducted to the entire chromosome sequence of the *E. coli* A17EC0191 strain (GenBank accession CP088822) using the COG database (1) and abundance of the coding sequences (CDSs) for the encoding protein of a function in each quarter was analyzed. Of a total of 4,967,354 bp of the *E. coli* A17EC0191 strain including 4,747 CDSs and each quarter included 1,241,838 bp.

References

1. Galperin MY, Makarova KS, Wolf YI, Koonin EV. 2015. Expanded microbial genome coverage and improved protein family annotation in the COG database. Nucleic Acids Res 43:D261-9.
